# Annual Meeting of the Erie County Medical Society

**Published:** 1882-02

**Authors:** 


### Society Reports.

---

### Erie County Medical Society—Annual Meeting.

The annual meeting of the Erie County Medical Society was held at the Medical College, Tuesday, January 10, the session opening at 10.30 A. M.

In the absence of the President, Dr. John Hauenstein, who is in Europe, the Vice-President, Dr. Thos. M. Johnson presided. Dr. A. M. Barker was at his post as Secretary.

The minutes of the June meeting were read and approved.

Dr. E. T. Dorland, as Chairman of the Committee on Membership, recommended the following for admission to the society: Drs. Clayton M. Daniels, Mary E. Runner, Edward Clark, Edward H. Ballou, John A. Hoffmeyer, Irving M. Snow, N. T. Keifer and C. G. Chaplain. The report was received and adopted.

Applications for membership were received from the following named gentlemen: Drs. A. H. Crawford, W. W. Potter, H. D. Ingraham, G. T. Brown, C. C. Frederick, G. W. York, C. A. McBeth, Walter D. Green, Floyd S. Crego, and M. D. Mann. They were referred to the Committee on Membership.

Action in regard to the application for membership of Dr. J. D. Bonnar was postponed until the semi-annual meeting in June next.

The Board of Censors presented the following report, which was received and adopted:

### REPORT OF THE BOARD OF CENSORS.

At the annual meeting, January 11th, 1881, the society instructed its Board of Censors:

First—To furnish the District-Attorney of this county with a list of the names of persons practicing in this county, medicine or surgery, who have failed to register their names in the County Clerk's office, in compliance with the new medical law of this State, and others who are known to them to practice in violation of the law, and urge their prosecution.

Second—To appeal through the legal firm of Messrs. Lewis & Rice to the Attorney General of this State for an order to bring the case of the "College of Physicians and Surgeons" of this city before the proper court, in order to determine the legality of the charter of this institution.

Your board would respectfully report: By the amendment passed by the last Legislature to the medical law of 1880, the time for registering was extended to the first of October last; we did not therefore deem it advisable to commence any action before that time.

In order to make up the list of names of persons practicing medicine illegally in this county, for reason of having failed to register, or an illegal registry, or practicing under cover of an illegal diploma or a diploma illegally obtained, the Board of Censors have appealed to the members of the medical profession of the county to aid them in securing the names and the proofs necessary to bring the cases before the grand jury. Your board regrets to state that the profession has shown no interest in the matter, and with but one or two exceptions, the appeal has been entirely ignored. Since the first of October Mr. Hatch, the District Attorney, has been busily engaged with important criminal cases and could not devote the necessary time for preparation to bring any of our cases before court except with extra legal help at our expense, and as the board had no such authority and no appropriation had been made for this purpose by the society, we deem it desirable to defer further action for the time being, not for the reason of "apathy towards the great interest in the vital questions entrusted to our charge," but simply for the want of funds.

In regard to the legal action brought against the institution that existed in this city under the name of "Buffalo College of Physicians and Surgeons," we beg leave to report:

The appeal for "an order" was granted by Attorney General Ward. The suit was brought before the Special Term of the Supreme Court, June, 1881, to test the question of validity of the charter of this institution. Judge Barker of the Supreme Court in this decision has overruled the demurrer of the defendants, "and gives leave to answer the complaint in twenty days on payment of costs; otherwise absolute judgment will be awarded to the plaintiff."

The very able, elaborate opinion of Judge Barker has been published in full in the Buffalo *Medical Journal* and the public press of the city. The defendants have appealed from the decision of Judge Barker to the General Term. The case is on the court calender for this term and will be argued on our side by Mr. Rice, who has now sole charge of our case. The whole expense for costs on our side will not exceed much the amount appropriated by the society. However large this expenditure for this purpose may seem, your board deem the money well and legitimately applied, if we take into consideration the great importance of the question involved, for the medical profession of the whole State.

The decision of Judge Barker, which will unquestionably be sustained by the General Term, will settle, beyond a doubt and for all time, the question whether this institution, that had been created in our city by some "philanthropists" and "humanitarians," a "benevolent, charitable, scientific and missionary society," and other kindred institutions in the State, can with a charter obtained under the law of 1848, "create and maintain a medical college, as such institutions are known to the laws of the State, and to confer honorary degrees and grant diplomas to its graduates," or whnther they shall be compelled by authority of the law to close their doors as bogus institutions.

EDWARD STORCK,
H. R. HOPKINS,
A. H. BRIGGS,
P. W. VAN PEYMA,
The Board of Censors.

BUFFALO, January 10th, 1882.

Dr. Storck, as Chairman of the committee regarding a Board of Health, reported progress.

The report of the Treasurer showed that some $300 had been expended during the past year, leaving only a balance of about $55 on hand. The report was received; a committee was appointed to audit it, and they subsequently reported it correct.

The report of the Librarian, Dr. J. B. Samo, was received and placed on file.

Dr. F. F. Hoyer presented a series of resolutions relative to the prosecution of persons practicing medicine or surgery contrary to the provisions of the new medical law. By vote of the society they were referred to the Board of Censors.

THE ELECTION OF OFFICERS

for the ensuing year resulted as follows: President, Dr. T. M. Johnson; Vice-President, Dr. S. E. S. H. Nott; Secretary, Dr. A. M. Barker; Treasurer, D. F. W. Abbott; Librarian, Dr. J. B. Samo.

Board of Censors—Drs. E. Storck, H. R. Hopkins, A. H. Briggs, P. W. Van Peyma, and F. F. Hoyer.

Committee on Membership—Drs. E. T. Dorland, Herman Mynter and T. M. Johnson.

Delegates to the State Medical Society—Drs. F. F. Hoyer, S. E. S. H. Nott, A. M. Barker, and H. R. Hopkins.

The annual address of the retiring President, Dr. John Hauenstein, was read by Dr. Bernard Bartow, and is published in full in this number of the Journal.

After transacting the usual routine business, the meeting adjourned.

